# Immune Checkpoint Inhibitor Outcomes in NSCLC Across Populations and Practice Settings

**DOI:** 10.1016/j.jtocrr.2026.100952

**Published:** 2026-01-05

**Authors:** Matthew Lee, Jialing Liu, Kari J. Teigen, Krishti Sabloak, Melissa Howell, Mario Gonzalez, Bassam Ghabach, David E. Gerber, Kalyani Narra, Mitchell S. von Itzstein

**Affiliations:** a UT Southwestern Medical School, University of Texas Southwestern Medical Center, Dallas, Texas; b Harold C. Simmons Comprehensive Cancer Center, University of Texas Southwestern Medical Center, Dallas, Texas; c Office of Clinical Research, JPS Health Network, Fort Worth, Texas; d Texas College of Osteopathic Medicine, Fort Worth, Texas; e IT Business Intelligence, JPS Health Network, Fort Worth, Texas; f JPS Oncology and Infusion Center, Fort Worth, Texas; g Burnett School of Medicine at Texas Christian University, Fort Worth, Texas; h Peter O’Donnell Jr. School of Public Health, University of Texas Southwestern Medical Center, Dallas, Texas; i Division of Hematology-Oncology, Department of Internal Medicine, University of Texas Southwestern Medical Center, Dallas, Texas

**Keywords:** ICI, Real-world, Non–small cell lung cancer, Safety-net

## Abstract

**Introduction:**

Immune checkpoint inhibitor (ICI) clinical trials generally enroll non-Hispanic White patients at academic or private practice settings. ICI outcomes at safety-net settings and across diverse populations remain limited.

**Methods:**

We conducted a retrospective study of patients with advanced NSCLC treated in a safety-net health care system and at an academic cancer center. We obtained clinical and demographic data from the electronic medical record. Kaplan-Meier estimates and Cox proportional hazards model were used to assess variables associated with survival.

**Results:**

A total of 408 patients were included. Compared with the academic center cohort (n = 213), the safety-net cohort (n = 195) was younger (35% versus 75% ≥ 65 y old; *p* < 0.001), had more racial and ethnic diversity (48% versus 73% non-Hispanic White; *p* < 0.001), and had more disadvantaged socioeconomic score (61 versus 24 median socioeconomic index; *p* < 0.001). After multivariable adjustment, patients receiving ICI as part of their treatment had improved survival compared with patients receiving chemotherapy alone (chemoradiation and ICI: adjusted hazard ratio [aHR] = 0.54; 95% confidence interval [CI]: 0.31–0.93; *p* = 0.03; chemotherapy and ICI: aHR = 0.44; 95% CI: 0.28–0.68; *p* < 0.001; ICI alone: aHR = 0.53; 95% CI: 0.30–0.91; *p* = 0.02). There was a near significant association with improved survival at academic practice setting (HR = 0.77; CI = 0.58–1.03; *p* = 0.08). There were no differences in survival according to race and ethnicity or socioeconomic status.

**Conclusion:**

In a diverse cohort across practice settings, receipt of ICI was associated with improved survival, regardless of facility type, race and ethnicity, or socioeconomic status. Efforts to provide ICI access to all eligible patients may improve outcomes.

## Introduction

Despite recent advances in therapy, NSCLC remains the leading cause of cancer-related mortality in the world.^1^^,^^2^ Before the introduction of immune checkpoint inhibitors (ICIs), the estimated 5-year survival for metastatic NSCLC was approximately 5%.^3^ ICIs have significantly affected the treatment landscape of NSCLC, with recent trials demonstrating 5-year survival rates between 20% and 30%.^3^, ^4^, ^5^, ^6^ Because of these promising results, ICIs have become widely adopted in clinical practice.^7^ However, these pivotal clinical trials were primarily conducted at academic or private practice institutions and lack representation from diverse populations.^8^^,^^9^ These limitations raise concerns about the generalizability of clinical trial findings to real-world settings, particularly across diverse racial, ethnic, and socioeconomically disadvantaged patient populations.

These under-represented patient groups disproportionately receive care in safety-net health care systems, which provide care to socioeconomically disadvantaged populations, including a large proportion of racial and ethnic minorities.^10^ Although up to 40% of patients with cancer receive their care at safety-net hospitals, there is a knowledge gap regarding the efficacy of ICIs in these settings.^11^ Patients at safety-net hospitals frequently face systemic challenges such as financial barriers, difficulties with transportation, and lack of caregiver support, which may affect survival outcomes compared with patients treated at academic centers.^12^ Further understanding of the interplay between race, ethnicity, socioeconomic status (SES), and practice setting is critical for the optimal implementation of ICIs into clinical practice.^13^

We, therefore, conducted a retrospective cohort study to evaluate treatment patterns and survival outcomes in a highly diverse population of patients with advanced NSCLC treated across practice settings, including a safety-net facility and an academic medical center. We included patients diagnosed with advanced NSCLC and not treated with ICIs for comparative analyses.

## Materials and Methods

### Study Procedures, Clinical Data Collection, and Characterization

We conducted a multicenter retrospective study in patients with advanced NSCLC at a safety-net cancer center (JPS Health Network [JPS]) and an academic cancer center (University of Texas Southwestern Medical Center [UTSW]). Both site locations are in North Texas; JPS in Fort Worth and UTSW in Dallas. This study was approved by the institutional review boards at both JPS (North Texas institutional review board 2063069-2) and UTSW (protocol #STU-2022-0760) and was exempted from requiring individual patient consent due to the retrospective nature of the study. Patients were included in our study population if they had stage IIIB, IIIC, or IV NSCLC. Staging criteria were determined from the American Joint Committee on Cancer seventh edition from January 1, 2017, to December 31, 2017, and American Joint Committee on Cancer eighth edition from January 1, 2018, to December 31, 2021.^14^^,^^15^ Patients were required to have received both their initial cancer diagnosis and first treatment decision at the same respective institution for inclusion. Therefore, patients diagnosed at other facilities and those diagnosed at JPS or UTSW who initiated treatment elsewhere were excluded. Patients with actionable driver alteration (*EGFR*, *ALK*, *ROS1*, *MET* exon 14 skipping, *BRAF*, NTRK, and *RET*) were excluded from our cohort because these patients are recommended to receive first-line treatment with molecularly targeted therapies. Patients whose tumors harbored *KRAS* or *HER2* mutations, for whom targeted therapy is generally given in the second-line setting, were included.

Data from the JPS cohort were obtained from the JPS Oncology Registry and manual review of the electronic medical record (EMR) by KS, MG, and KN, and the UTSW cohort was obtained from the UTSW Institutional Tumor Registry and manual EMR review by ML and MVI. Data obtained through manual EMR review included cancer stage, first-line treatment approach, death date, last known follow-up date, molecular testing including PD-L1 status, smoking status and history, and Eastern Cooperative Oncology Group performance status. At both sites, the cohort included patients diagnosed with NSCLC between January 1, 2017, and December 31, 2021. We selected this period because (1) first-line use of ICI was established as standard therapy for NSCLC lacking actionable genomic alterations, and (2) it provides adequate follow-up to evaluate survival outcomes. Demographic data, including age, sex, race, ethnicity, and SES, were obtained from tumor registry. SES was inferred using the patient’s zip code and the previously validated Health Equity Index from Healthy North Texas based on zip code, scored from 1 to 100^16^^,^^17^ (higher scores reflect more disadvantaged SES). Clinical data included cancer stage, histology, PD-L1 expression (categorized as < 1%, 1%–49%, ≥ 50%, or not tested), smoking status (former, current, or never) and pack-years, body mass index (collected because higher levels are associated with improved survival, categorized as underweight < 18.4, normal weight 18.5–24.9, overweight 25–29.9, obese > 30),^18^ Charlson comorbidity index, and first-line treatment approach, a metric collected by tumor registries. First-line treatments were categorized as chemotherapy alone, ICI alone, chemoradiation followed by ICI, and combination chemotherapy with ICI. Patients were also included if the treatment approach was no systemic cancer therapy (either patient or clinician decision). The overall survival (OS) end point was defined as the time from the date of diagnosis to the date of death, and patients without a death date were censored at the last known follow-up.

### Statistical Analysis

The demographic and clinical characteristics were summarized by the academic center and the safety-net cohorts using descriptive statistics. Patient characteristics were compared using the chi-square or Fisher’s exact test for categorical variables and the Wilcoxon ranked sum test for continuous variables. The Kaplan-Meier (KM) method was used to estimate OS probabilities by race-ethnicity groups in the combined cohort, stratified by site, and in a subgroup of patients treated with ICIs. To assess differences in survival distributions across race-ethnicity groups, a mixed-effects Cox proportional hazard model was used for the combined cohort to account for site-level clustering, whereas standard Cox proportional hazard models were applied to the individual academic center and the safety-net cohorts. To identify patient characteristics associated with survival outcome in the combined cohort, we first used a univariate Cox proportional hazard model to select potentially associated factors. Variables with *p* less than 0.2 in the univariate analyses were then included in a multivariable Cox proportional hazard model. Both unadjusted hazard ratios (HRs) and adjusted hazard ratios (aHRs) with 95% confidence intervals (CIs) were reported. All statistical analyses were performed using R (version [v.] 4.1.3) and SAS v. 9.4 (Cary, NC). A two-sided *p* value less than 0.05 was considered statistically significant.

## Results

A total of 408 patients were included in the overall cohort, with 195 patients from the safety-net site and 213 patients from the academic site (Table 1). Patients at the safety-net site tended to be younger with 35% aged 65 years or older compared with 75% at the academic site (*p* < 0.001). The safety-net site was more diverse; 34% Black, 48% non-Hispanic White, 13% Hispanic, and 6% Asian, compared with 19% Black, 73% non-Hispanic White, 6% Hispanic, and 2% Asian patients at the academic site (*p* < 0.001). Patients at the safety-net site had a more disadvantaged SES with a median socioeconomic index score of 61 compared with 24 at the academic site (*p* < 0.001). Notably, 82% of patients in the safety-net site had stage IV disease compared with 69% at the academic site (*p* = 0.005). Treatment patterns differed between sites. Patients at the safety-net site were less likely to receive systemic therapy (56% compared with 35% at the academic site [*p* < 0.001]). At the academic site, among the patients who received systemic therapy, 65% received ICI as part of their treatment, compared with 55% at the safety-net site (*p* = 0.17).

**Table 1:** Baseline Clinical and Demographic Characteristics

Characteristic, n (%)	Overall (N = 408)	Academic Center (n = 213)	Safety Net (n = 195)	*p* Value
Age, y^a^				
< 65	180 (44%)	54 (25%)	126 (65%)	< 0.001
≥ 65	228 (56%)	159 (75%)	69 (35%)	
Sex^a^				
Male	243 (60%)	127 (60%)	116 (59%)	1
Female	165 (40%)	86 (40%)	79 (41%)	
Race-ethnicity^a^				
Non-Hispanic Black	107 (26)	41 (19)	66 (34)	< 0.001
Non-Hispanic White	248 (61)	155 (73)	93 (48)	
Hispanic	38 (9)	13 (6)	25 (13)	
Asian	15 (4)	4 (2)	11 (6)	
Cancer stage^a^				
IIIB–C	100 (25%)	65 (31%)	35 (18%)	0.005
IV	308 (75%)	148 (69%)	160 (82%)	
Cancer treatment^b^				
Chemotherapy	65 (16%)	36 (17%)	29 (15%)	< 0.001
Chemoradiation	21 (5%)	12 (6%)	9 (5%)	
Chemoradiation + ICI	34 (8%)	28 (13%)	6 (3%)	
Chemotherapy + ICI	70 (17%)	44 (21%)	26 (14%)	
ICI	34 (8%)	19 (9%)	15 (8%)	
No systemic therapy	181 (45%)	74 (35%)	107 (56%)	
Unknown	3	0	3	
Histology^a^				
Squamous	137 (34%)	70 (33%)	67 (34%)	0.83
Nonsquamous	271 (66%)	143 (67%)	128 (66%)	
PD-L1^a^				
Not performed	153 (38%)	86 (40%)	67 (34%)	0.06
< 1%	76 (19%)	48 (23%)	28 (14%)	
1%–49%	93 (23%)	47 (22%)	46 (24%)	
≥ 50%	86 (21%)	32 (15%)	54 (28%)	
Smoking status^a^				
Former	230 (59%)	138 (65%)	92 (52%)	0.003
Current	121 (31%)	51 (24%)	70 (40%)	
Never	38 (10%)	24 (11%)	14 (8%)	
Unknown	19	0	19	
Pack year^c^				
Median (IQR)	30 (13, 50)	40 (15, 53)	20 (8, 30)	< 0.001
Unknown	77	8	69	
BMI, kg/m^2^^a^				
< 18.4	48 (13%)	15 (8%)	33 (17%)	0.001
18.5–24.9	160 (43%)	66 (37%)	94 (48%)	
25–29.9	90 (24%)	55 (31%)	35 (18%)	
> 30	74 (20%)	42 (24%)	32 (16%)	
Unknown	36	35	1	
Charlson comorbidity score^c^				
Median (IQR)	9 (5, 12)	10 (5, 13)	8 (5, 11)	< 0.001
Socioeconomic status score (health equity index)^c^				
Median (IQR)	38 (17, 72)	24 (9, 63)	61 (26, 91)	< 0.001
Unable to calculate^d^	30	27	3	

BMI, body mass index; ICI, immune checkpoint inhibitor; IQR, interquartile range.

a Chi-square test.

b Fisher’s exact test.

c Wilcoxon ranked sum test.

d Health equity index from Healthy North Texas (higher scores reflect worse socioeconomic status).

**Figure 1:**
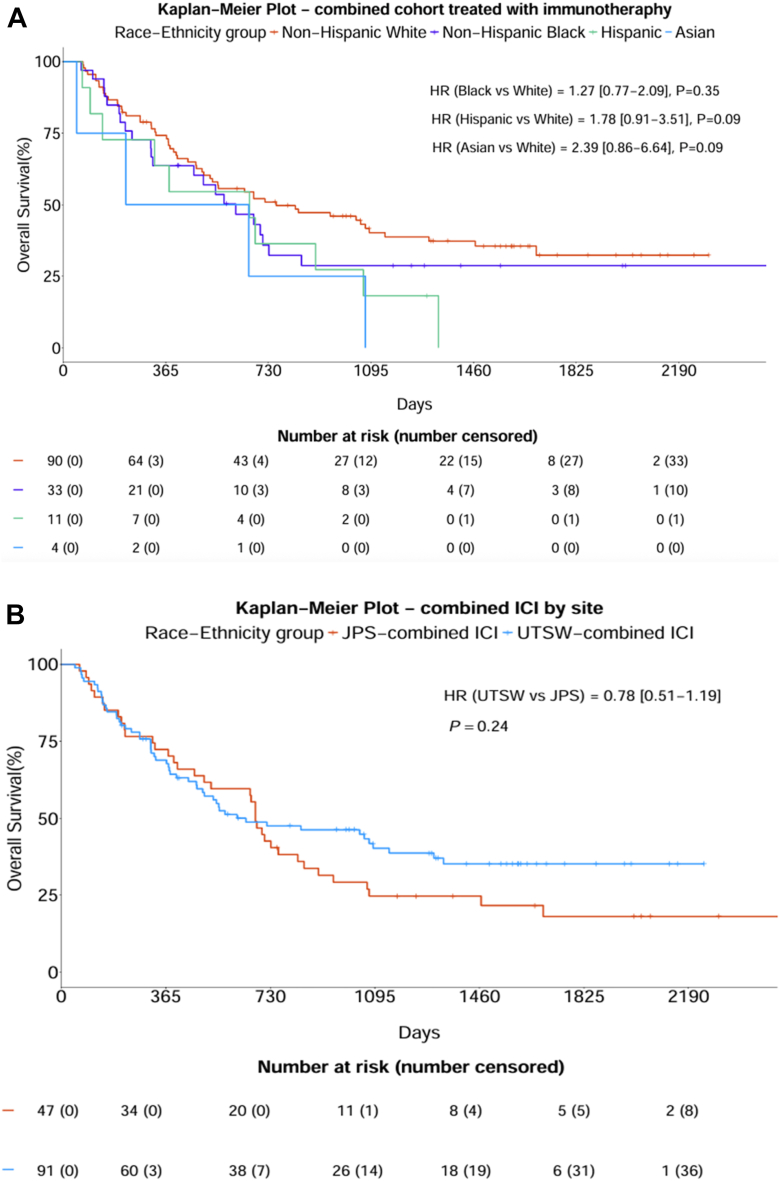
Overall survival in ICI-treated patients in the overall cohort (n = 138) (*A*) according to race and ethnicity and (*B*) according to practice setting. HR, hazard ratio; ICI, immune checkpoint inhibitor; JPS, JPS Health Network; UTSW, University of Texas Southwestern Medical Center.

**Figure 2:**
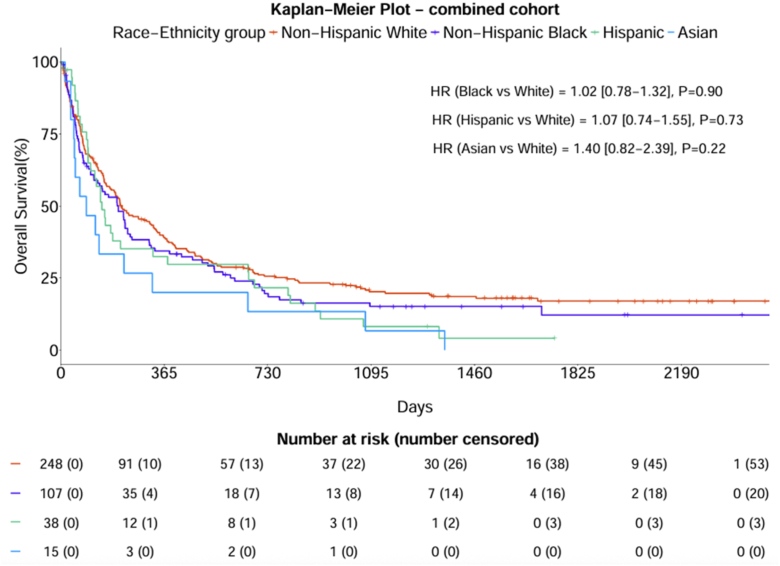
Overall survival according to race and ethnicity in the overall cohort. HR, hazard ratio.

In the subcohort of patients treated with ICI, there were no differences in survival according to race or ethnicity (Fig. 1*A*). Furthermore, there were no differences in survival according to practice setting in ICI-treated patients (Fig. 1*B*). Overall, there were no differences in survival between racial and ethnic subgroups (Fig. 2). At the academic site, Hispanic and Asian patients had inferior OS with unadjusted HRs of 1.96 (95% CI: 1.08–3.56) and 3.29 (95% CI: 1.20–9.00) (Supplementary Fig. 1A). At the safety-net site, there were no differences in survival between racial and ethnic subgroups (Supplementary Fig. 1B). In the univariate survival analysis, the academic site had improved survival (HR = 0.59; 95% CI: 0.47–0.73; *p* < 0.001) (Supplementary Table 1). After adjusting for covariates in the multivariable analysis, there was no longer a statistically significant survival difference between the JPS and UTSW sites, although there was a near significant trend (*p* = 0.08) (Table 2). In addition, the multivariable analysis revealed significantly worse outcomes for patients with stage IV disease (aHR = 2.04; 95% CI: 1.46–2.83; *p* < 0.001) compared with patients with stage IIIB-C and patients who received no cancer treatment (aHR = 2.73; 95% CI: 1.91–3.91; *p* < 0.001) compared with those who received chemotherapy alone. Meanwhile, patients who received chemotherapy combined with ICI (aHR = 0.44; 95% CI: 0.28–0.68; *p* < 0.001), chemoradiation followed by ICI (aHR = 0.54; 95% CI: 0.31–0.93; *p* = 0.03), and ICI alone (aHR = 0.53; 95% CI: 0.30–0.91; *p* = 0.02) had improved survival compared with patients who received chemotherapy alone.

**Table 2:** Multivariable Survival Analysis of the Combined Cohort

Characteristic	Adjusted Hazard Ratio (95% CI)	*p* Value
Site		
Safety net	Reference	-
Academic center	0.77 (0.58–1.03)	0.08
Race-ethnicity		
Non-Hispanic White	Reference	-
Non-Hispanic Black	1.05 (0.77–1.43)	0.75
Hispanic	1.07 (0.66–1.74)	0.78
Asian	1.29 (0.71–2.35)	0.41
Sex		
Male	Reference	-
Female	0.71 (0.54–0.94)	0.02
Cancer stage		
IIIB–C	Reference	-
IV	2.04 (1.46–2.83)	< 0.001
Cancer treatment		
Chemotherapy	Reference	-
Chemotherapy + radiation	0.78 (0.41–1.47)	0.45
Chemotherapy + radiation + ICI	0.54 (0.31–0.93)	0.03
Chemotherapy + ICI	0.44 (0.28–0.68)	< 0.001
ICI	0.53 (0.30–0.91)	0.02
No systemic therapy	2.73 (1.91–3.91)	<0.001
Histology		
Squamous	Reference	-
Nonsquamous	0.64 (0.48–0.86)	0.002
PD-L1		
Not performed	Reference	-
< 1%	1.06 (0.73–1.56)	0.75
1%–49%	0.97 (0.68–1.39)	0.88
≥ 50%	0.84 (0.56–1.26)	0.40
Smoking status		
Former	Reference	-
Current	0.97 (0.73–1.30)	0.84
Never	1.27 (0.80–2.03)	0.32
BMI, kg/m^2^		
< 18.4	Reference	-
18.5–24.9	0.64 (0.43–0.95)	0.03
25–29.9	0.67 (0.43–1.04)	0.08
> 30	0.89 (0.57–1.40)	0.62
Socioeconomic status score (health equity index)	1.00 (0.99–1.00)	0.14

BMI, body mass index; CI, confidence interval; ICI, immune checkpoint inhibitor.

## Discussion

We conducted a real-world study evaluating treatment patterns and outcomes of patients with advanced NSCLC treated at a safety-net hospital and an academic cancer center. Our study revealed that Black, Hispanic, Asian, and non-Hispanic White patients have comparable survival in advanced NSCLC, including in the subgroup of the cohort that received ICIs. Furthermore, after multivariate adjustment, there were no survival differences between an academic and a safety-net practice setting, although there was a trend favoring improved survival at the academic setting. Given the challenges faced at safety-net facilities, this is an encouraging finding that is impressive considering the likelihood of unmeasured confounders (such as toxicity or compliance) that could not be adjusted for in this retrospective nonrandomized analysis. The most notable variable independently associated with worse survival was lack of receipt of systemic therapy, which occurred in 56% of patients treated at the safety-net facility. Furthermore, receiving ICI as part of the treatment approach was associated with substantially improved survival compared with chemotherapy alone.

To date, there are few studies evaluating the outcomes of different racial and ethnic patients treated with ICIs. Of the existing studies, findings suggest that Black patients have similar or even improved survival when treated with ICIs compared with non-Hispanic White patients.^19^, ^20^, ^21^ For instance, a retrospective cohort study at the Veterans Health Administration found that Black patients had comparable survival outcomes and lower rates of immune-related adverse events compared with White patients.^19^ Other retrospective studies reported that OS and progression-free survival were similar across different racial and ethnic groups, including Black and Hispanic patients.^20^^,^^21^ The diverse racial and ethnic backgrounds in the safety-net cohort allowed us to further explore the efficacy of ICIs in these patients. Importantly, we evaluated SES as a covariate, which has previously been suggested to underlie differences in cancer-specific survival outcomes with minority patients.^22^ The Asian patients had substantially inferior survival at the academic site, although the extremely small numbers in this subgroup make this finding of unclear significance. One potential explanation is that the exclusion of *EGFR* mutation-positive patients (a group in which Asian patients are typically highly represented) left a remaining Asian subgroup with a poorer prognosis.

We also revealed that SES, as approximated by the Health Equity Index, was not an independent predictor of survival in our combined cohort. Although studies have demonstrated that patients with metastatic NSCLC with low SES have worse OS, this disparity appears to disappear in patients treated with ICIs.^23^^,^^24^ Taken together, these findings suggest that once initiated, ICIs provide comparable survival benefits regardless of SES, race and ethnicity, or practice setting. An obvious key focus should be on improving access to ICIs and diagnosing cancer earlier to increase the likelihood that the patients can receive timely treatment before declining performance status.

Notably, patients at the safety-net facility were more likely to have stage IV disease at diagnosis compared with UTSW patients. Studies have demonstrated that Black patients and patients of lower SES are more frequently diagnosed with stage IV NSCLC.^25^^,^^26^ One potential reason could be that patients with lower income are less likely to complete recommended lung cancer screening, thereby reducing the chance to diagnose the disease at an earlier stage.^27^ Furthermore, patients presenting to safety-net facilities may not seek care for lung cancer symptoms because of financial or socioeconomic barriers until later in the disease course, so they tend to present to emergency departments as opposed to being seen at in outpatient clinics for lung cancer workup. Furthermore, patients at the academic center had slightly higher Charlson Comorbidity Index scores compared with safety-net site patients. This difference is at least partially driven by the older age of the academic site patients because age above or equal to 80 years adds 4 points to the score.^28^ Literature suggests that patients with higher Charlson Comorbidity Index scores present with earlier stage NSCLC compared with those with lower scores, possibly because they engage with the health care system more frequently.^29^

Despite being more likely to have stage IV disease, patients at the safety-net sites were less likely to receive immunotherapy or combined chemoimmunotherapy. These findings align with prior research demonstrating that patients with low income, non-White race, or inadequate insurance coverage are less likely to receive ICIs.^23^^,^^24^ The higher rate of no systemic therapy at the safety-net site likely reflects systemic barriers, including financial challenges and logistical issues such as transportation to receive care. Other reasons include needing to start systemic treatment in the inpatient setting for symptoms and biomarker testing not readily available for oncologists to inform treatment decisions. Prior studies reveal that up to half of patients with NSCLC do not receive systemic therapy, consistent with our findings at the safety-net site.^30^

Our study has several limitations. First, this is a retrospective study, which introduces the potential for selection bias. We also lacked PD-L1 testing in a large subset of patients. Furthermore, the patients received varying treatment approaches incorporating ICI, likely reflecting stage, PD-L1 status, and performance status, rendering it difficult to isolate the effect of ICI on outcomes. Despite including two centers, the overall sample size for the study is relatively modest, and this is reflected in the limited number of patients from racial and ethnic minority backgrounds. It is standard practice at both institutions to refer all patients with advanced lung cancer to palliative care, but this variable was not included in the analysis of this study. Because Eastern Cooperative Oncology Group performance status is rarely captured completely and accurately in retrospective clinical studies, we did not include this variable in our analyses, and this is a limitation because it can confound survival analyses and treatment selection. Furthermore, we did not have data on later lines of therapy, which can affect clinical outcomes. The major strengths of the study include the multicenter analysis including an academic and safety net facility, detailed and comprehensive clinical and demographic data including socioeconomic index, and diverse patient population, with considerable representation from racial and ethnic minorities and socioeconomically disadvantaged groups.

In conclusion, patients treated within the safety-net setting were younger, more racially and ethnically diverse, had more disadvantaged socioeconomic index scores, but a lower Charlson Comorbidity Index compared with patients treated within the academic setting. OS outcomes were comparable regardless of race, ethnicity, SES, including in ICI-treated patients. Lack of receipt of systemic therapy was independently associated with worse survival. Administration of ICI as part of the treatment approach was associated with improved survival regardless of other characteristics. Efforts to increase access to ICIs may further improve outcomes.

## CRediT Authorship Contribution Statement

**Matthew Lee:** Writing – review & editing, Data collection, Data curation, Writing – original draft, Investigation.

**Jialing Liu:** Writing – review & editing, Formal analysis, Data curation, Visualization, Software.

**Kari J. Teigen:** Writing – review & editing, Formal analysis, Data curation.

**Krishti Sabloak:** Writing – review & editing, Investigation.

**Melissa Howell:** Writing – review & editing, Investigation.

**Mario Gonzalez:** Writing – review & editing, Investigation.

**Bassam Ghabach:** Writing – review & editing, Investigation.

**David E. Gerber:** Writing – review & editing, Conceptualization, Supervision, Investigation.

**Mitchell S. von Itzstein:** Writing – review & editing, Data collection, Data curation, Writing – original draft, Investigation, Supervision, Project administration, Conceptualization.

**Kalyani Narra:** Writing – review & editing, Data collection, Data curation, Writing – original draft, Investigation, Supervision, Project administration, Conceptualization.

## Data Availability

Deidentified data will be available on reasonable request to the corresponding author.

## Disclosure

The authors declare no conflict of interest.

## Footnotes

Note: To access the supplementary material accompanying this article, visit the online version of the *JTO Clinical and Research Reports* at www.jtocrr.org and at https://doi.org/10.1016/j.jtocrr.2026.100952.
