# Supplementary material for: Immune Checkpoint Inhibitor Outcomes in NSCLC Across Populations and Practice Settings
Source: JTO Clin Res Rep. 2026 Jan 5;7(4):100952. doi: 10.1016/j.jtocrr.2026.100952 (PMC12992504; doi:10.1016/j.jtocrr.2026.100952)
Supplement: Supplemental Table 1 [file mmc1.docx]

**Supplemental Table 1.** Univariate survival analysis of the combined cohort.

| **Characteristic** | **Unadjusted Hazards Ratio (95% CI)** | ***P* value** |
| --- | --- | --- |
| Site^*^ |  |  |
| Safety-net | Reference | - |
| Academic center | 0.59 (0.47, 0.73) | < .001 |
| Race-ethnicity^*^ |  |  |
| Non-Hispanic White | Reference | - |
| Non-Hispanic Black | 1.15 (0.89, 1.48) | .27 |
| Hispanic | 1.30 (0.90, 1.86) | .16 |
| Asian | 1.77 (1.05, 2.99) | .03 |
| Age, year |  |  |
| <65 | Reference | - |
| ≥65 | 0.96 (0.77, 1.19) | .69 |
| Sex^*^ |  |  |
| Male | Reference | - |
| Female | 0.81 (0.65, 1.02) | .07 |
| Cancer stage^*^ |  |  |
| IIIB-C | Reference | - |
| IV | 1.65 (1.27, 2.14) | < .001 |
| Cancer treatment^*^ |  |  |
| Chemotherapy | Reference | - |
| Chemotherapy + radiation | 0.73 (0.42, 1.28) | .27 |
| Chemotherapy + radiation + immunotherapy | 0.47 (0.28, 0.79) | .004 |
| Chemotherapy + immunotherapy | 0.54 (0.37, 0.80) | .002 |
| Immunotherapy | 0.61 (0.38, 0.97) | .04 |
| No systemic therapy | 3.15 (2.31, 4.31) | < .001 |
| Histology^*^ |  |  |
| Squamous | Reference | - |
| Non-squamous | 0.71 (0.57, 0.89) | .003 |
| PD-L1^*^ |  |  |
| Not performed | Reference | - |
| <1% | 0.99 (0.73, 1.33) | .93 |
| 1-49% | 0.72 (0.54, 0.97) | .03 |
| >=50% | 0.75 (0.56, 1.01) | .06 |
| Smoking status^*^ |  |  |
| Former | Reference | - |
| Current | 1.29 (1.01, 1.65) | .04 |
| Never | 0.98 (0.67, 1.43) | .90 |
| Pack year | 1.00 (0.99, 1.00) | .70 |
| BMI, kg/m^2*^ |  |  |
| <18.4 | Reference | - |
| 18.5-24.9 | 0.69 (0.48, 0.97) | .04 |
| 25-29.9 | 0.58 (0.40, 0.86) | .01 |
| >30 | 0.65 (0.44, 0.96) | .03 |
| Charlson comorbidity score | 1.00 (0.97, 1.02) | .78 |
| Socioeconomic status^*^ score(Health equity index) | 1.00 (1.00, 1.01) | .06 |
| * Variables included in the multivariable survival analysis. | | |
